# Comparison of Facial Muscle Activation Patterns Between Healthy and Bell’s Palsy Subjects Using High-Density Surface Electromyography

**DOI:** 10.3389/fnhum.2020.618985

**Published:** 2021-01-12

**Authors:** Han Cui, Weizheng Zhong, Zhuoxin Yang, Xuemei Cao, Shuangyan Dai, Xingxian Huang, Liyu Hu, Kai Lan, Guanglin Li, Haibo Yu

**Affiliations:** ^1^Department of Acupuncture and Moxibustion, Shenzhen Traditional Chinese Medicine Hospital, Shenzhen, China; ^2^CAS Key Laboratory of Human-Machine Intelligence-Synergy Systems, Shenzhen Institutes of Advanced Technology, Chinese Academy of Sciences, Shenzhen, China; ^3^The Fourth Clinical Medical College, Guangzhou University of Chinese Medicine, Shenzhen, China

**Keywords:** facial muscle, facial movement, Bell’s palsy, facial nerve, high-density surface EMG

## Abstract

Facial muscle activities are essential for the appearance and communication of human beings. Therefore, exploring the activation patterns of facial muscles can help understand facial neuromuscular disorders such as Bell’s palsy. Given the irregular shape of the facial muscles as well as their different locations, it should be difficult to detect the activities of whole facial muscles with a few electrodes. In this study, a high-density surface electromyogram (HD sEMG) system with 90 electrodes was used to record EMG signals of facial muscles in both healthy and Bell’s palsy subjects when they did different facial movements. The electrodes were arranged in rectangular arrays covering the forehead and cheek regions of the face. The muscle activation patterns were shown on maps, which were constructed from the Root Mean Square (RMS) values of all the 90-channel EMG recordings. The experimental results showed that the activation patterns of facial muscles were distinct during doing different facial movements and the activated muscle regions could be clearly observed. Moreover, two features of the activation patterns, 2D correlation coefficient (corr2) and Centre of Gravity (CG) were extracted to quantify the spatial symmetry and the location of activated muscle regions respectively. Furthermore, the deviation of activated muscle regions on the paralyzed side of a face compared to the healthy side was quantified by calculating the distance between two sides of CGs. The results revealed that corr2 of the activated facial muscle region (classified into forehead region and cheek region) in Bell’s palsy subjects was significantly (*p* < 0.05) lower than that in healthy subjects, while CG distance of activated facial region in Bell’s palsy subjects was significantly (*p* < 0.05) higher than that in healthy subjects. The correlation between corr2 of these regions and Bell’s palsy [assessed by the Facial Nerve Grading Scale (FNGS) 2.0] was also significant (*p* < 0.05) in Bell’s palsy subjects. The spatial information on activated muscle regions may be useful in the diagnosis and treatment of Bell’s palsy in the future.

## Introduction

The face is an important part of the appearance of the human body. Additionally, the superficial geometric changes in the face are produced by the facial muscle (Cattaneo and Pavesi, 2014). Facial muscles are innervated by the facial nerve, which emerges from the Pons of the brainstem, travels through the facial canal in the temporal bone, and exits the skull at the stylomastoid foramen (Peitersen, 2002). As soon as the facial nerve exits the stylomastoid foramen, it splits into five terminal branches, namely the temporal, zygomatic, buccal, marginal (or mandibular), and cervical branches (Cattaneo and Pavesi, 2014). These branches anatomize with each other within the parotid gland and form a variably intricate plexus (Captier et al., 2005). Moreover, the facial muscle is unique in the human musculoskeletal system owing to its heterogeneity among various muscles on the face as well as within the same muscle (Cattaneo and Pavesi, 2014). Furthermore, it was reported that there exist considerable differences in the facial muscles of various individuals (D’Andrea and Barbaix, 2006). Consequently, this complex and diverse anatomical structure of the facial neuromuscular system enables the face to perform a vast range of facial movements. The various facial movements are in turn essential in daily life and affect a wide range of human behaviors including feeding, speech, and communication (Cattaneo and Pavesi, 2014). Therefore, a quantified investigation of the spatial activation patterns of facial muscles under different facial movements is important in understanding the recruitment of complex facial muscles. Also, it can provide a basis for understanding abnormalities in the facial neuromuscular system including Bell’s palsy.

Bell’s palsy is one of the most common causes of unilateral facial neuromuscular impairment in the world (Gilden, 2004). It is caused by injury of the facial nerve which is the seventh cranial nerve and innervates 17 pairs of facial muscles (Peitersen, 2002). The average annual incidence of Bell’s palsy was reported to be 20–35 per 100,000 people (Hauser et al., 1971; Rowlands et al., 2015) and it accounts for about 60–75% of all cases of unilateral facial paralysis (Adour et al., 1978). Moreover, about 29% of Bell’s palsy patients eventually exhibit mild to severe deficits (Glass and Kallirroi, 2014). Bell’s palsy has various consequences, including distortion of facial symmetry, incomplete eyelid closure, hemifacial spasms, synkinesis, permanent loss of taste, and gustatory lacrimation (Peitersen, 1982; Gilden, 2004). Therefore, patients manifesting such symptoms need careful examination to ascertain which facial muscle was impaired as well as the extent of impairment.

Currently, grading scales are the most commonly used methods for evaluating facial motor function. These grading scales score the facial motor function in either overall or regional way. For example, House–Brackmann Scale [also called the Facial Nerve Grading Scale (FNGS)] is a six-grade (I–VI) system that offers a gross evaluation of facial motor function (House and Brackmann, 1985; Berg et al., 2004; Vrabec et al., 2009). Yanagihara Scale and Sunnybrook Scale are regional grading systems which measure aspects of function in different facial muscles (House and Brackmann, 1985; Berg et al., 2004; Vrabec et al., 2009), and the latest developed Facial Nerve Grading Scale 2.0 (FNGS 2.0) combines regional scores with gross scores in the evaluation of facial function (House and Brackmann, 1985; Berg et al., 2004; Vrabec et al., 2009). However, although these scales are convenient for clinical use, the estimations are highly dependent on a clinician’s subjective observation (Rickenmann et al., 1997). Objective profiling of facial neuromuscular activities, employs electrophysiological testing methods, such as Electroneurography (EnoG) and Electromyography (EMG; Grosheva et al., 2008; Ozgur et al., 2010). The EnoG method uses maximal electrical stimulation to evoke the facial Compound Muscle Action Potential (CMAP). The ratio of CMAP on the paralyzed side of the healthy side then reflects degeneration of the facial nerve in the affected region (Gilden, 2004). However, the prognostic value of EnoG is usually affected by the position of the recording electrode due to the heterogeneity of facial muscles and the facial nerve (Engstrm et al., 2000; Kim et al., 2016).

EMG examinations are also utilized in diagnosing Bell’s palsy or monitoring functional changes during treatment of the condition (On et al., 2007; Grosheva et al., 2008; Han et al., 2015). However, facial EMG recordings using only two to six pairs of bipolar electrodes during the resting state or voluntary movement cannot acquire complete information on the complex activation of facial muscles (On et al., 2007; Grosheva et al., 2008; Han et al., 2015). Therefore, the high-density surface EMG (HD sEMG) technique may be an optimal method of comprehensively recording the activation of facial muscles for two reasons: (i) it is noninvasive so multiple electrodes can be attached to the surface of the face to increase the recording area; and (ii) the small electrode size (usually under 10 mm) and small Inter-electrode Distance (IED; usually under 15 mm) can reduce the crosstalk of EMG signals and increase the accuracy of recording (Winter et al., 1994; Hoffman and Strick, 1999). HD sEMG has been used in characterizing the distribution of muscle electrical activities in various parts of the body, including the forearm, shoulder, and laryngeal muscles (Dick et al., 2012; Zhu et al., 2017; Bracken et al., 2019; Dai and Hu, 2019). From the physiological perspective point of view, the advantage of HD sEMG lies in the non-invasive acquisition of detailed spatial neuromuscular information (Dick et al., 2012). Conventional sEMG techniques comprising a bipolar signal on one muscle is of little or no use in clinical diagnosis since this signal is a compound signal comprising EMG signals from different parts of the muscle (Pullman et al., 2000). In contrast, HD sEMG can extract neuromuscular information on a motor unit (MU) level since its high spatial resolution in recording EMG signals. It is in the MU level, HD sEMG technique can provide useful pathological information like the intramuscular needle EMG technique provided, and needle EMG technique is the standard method for assessing neuromuscular diseases (Gea Drost et al., 2006). For example, muscle fiber conduction velocity (MFCV) extracted from HD sEMG signals is an important feature in the assessment of muscle fatigue and motor neuron disease (Wood et al., 2001; Schillings et al., 2004). Innervation zone localization based on the HD sEMG technique can be applied for optimizing the botuline toxin injection (Walker et al., 2015). Thus, HD sEMG provides both classic and new information regarding the neuromuscular system in health and disease. Also, HD sEMG is particularly advantageous concerning extracting the activation patterns of muscles with complex anatomical structures (Gallina and Botter, 2013; Hu et al., 2015; Dai and Hu, 2019). Moreover, a few studies exist on the activation patterns of facial muscles using multiple channels surface EMG (Lapatki et al., 2003, 2006; Schumann et al., 2010) although some only examined the lower part of the facial muscles (Lapatki et al., 2003, 2006). In another example, Schumann et al. (2010) used 48 electrodes to characterize the activation pattern of facial muscles, although the placement of electrodes was empirical (Schumann et al., 2010) and may have been affected by inter-individual variations. Furthermore, these studies only examined healthy subjects, yet patients such as those with Bell’s palsy may exhibit different degrees of shifting in the spatial distribution of facial muscle activation.

The purpose of this study was to evaluate the activation patterns of all the facial muscles without prior knowledge of facial anatomy. Therefore, facial activation maps at different facial movements were obtained using HD sEMG and visualized by constructing the Root Mean Square (RMS) of the EMG signals. The facial movements included Raising Eyebrows (RE), Closing Eyes (CE), Bulging Cheek (BC), Grinning (GR), Pouting (PO), and Wrinkling Nose (WN). The movements were aimed at selectively activating muscles localized in different parts of the face. It is noteworthy that these facial movements are frequently used in the assessment of Bell’s palsy (Gilden, 2004). Moreover, symmetry in the activation of facial muscles during different movements was quantified by calculating the two-dimensional correlation coefficient between the left and right sides of the activation maps. Thereafter, differences in the activation symmetry of healthy and Bell’s palsy subjects were compared. Furthermore, the Centre of Gravity (CG) of the activated regions (segmented by the threshold) was extracted from the activation map to quantitively present the location of the activated regions. Finally, the study evaluated the correlation between the position of CG and Bell’s palsy. The findings accurately depicted the activation of muscles during basic facial movements in both healthy and Bell’s palsy subjects. This, therefore, provides further information on the complex activation of facial muscles under normal and pathological conditions such as Bell’s palsy.

## Materials and Methods

### Subjects

In this study, a total of 20 subjects was recruited, including 10 Bell’s palsy and 10 healthy individuals. The 10 Bell’s palsy subjects (four male, six female), aged between 24 and 67 years were recruited from the outpatient department of the Shenzhen Traditional Chinese Medicine Hospital. Among the subjects, six were affected on the left side of the face while four were affected on the right side. Moreover, the 10 healthy subjects (seven male, three female), aged between 24 and 65 years were among the employees of Shenzhen Traditional Chinese Medicine Hospital. The study ensured that the included Bell’s palsy subjects had no history of central or peripheral nerve diseases or injuries except for Bell’s palsy. Afterward, clinical assessments of the subjects’ degree of Bell’s palsy were conducted by an experienced doctor (more than 10 years of clinical experience on Bell’s palsy) using the FNGS 2.0, before the experiment (Vrabec et al., 2009). The FNGS 2.0 assesses different regions of the face and therefore can provide more information on regional facial movement compared to a single global score. Demographic information on the 20 subjects is presented in Table 1.

**Table 1:** The demographic information of all the subjects.

ID	Gender	Age	Paretic	FNGS	FNGS	FNGS	FNGS	FNGS
		(years)	side	score (brow)	score (eye)	score (NLF)	score (oral)	score (total)
1	F	62	L	5	2	6	6	19
2	F	44	L	1	1	1	2	5
3	M	49	R	6	5	6	6	23
4	M	24	L	5	2	2	3	14
5	F	38	L	6	4	6	6	22
6	F	67	R	3	3	3	3	12
7	F	58	R	2	2	3	2	9
8	M	34	L	6	3	6	6	21
9	M	63	R	2	2	2	2	8
10	F	28	L	3	2	3	3	11
11	M	27	–	1	1	1	1	4
12	F	65	–	1	1	1	1	4
13	F	24	–	1	1	1	1	4
14	F	39	–	1	1	1	1	4
15	M	46	–	1	1	1	1	4
16	M	37	–	1	1	1	1	4
17	M	30	–	1	1	1	1	4
18	M	23	–	1	1	1	1	4
19	M	26	–	1	1	1	1	4
20	M	27	–	1	1	1	1	4

*F, female; M, male; L, left; R, right; FNGS, facial nerve grading scale; NLF, nasolabial fold*.

This study was approved by the Institutional Review Board of Shenzhen Traditional Chinese Medicine Hospital (Approval Number: 2017-05) and was performed following the guidelines of the Declaration of Helsinki. Additionally, all the subjects personally gave written informed consent before the experiment.

### Instrumentation

**Figure 1:**
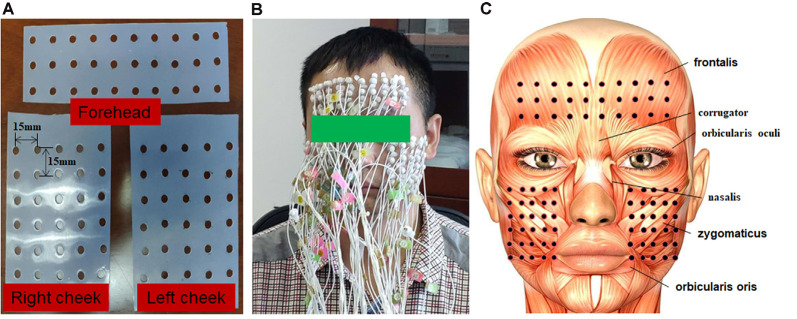
The experimental setup of the electrode grids. **(A)** Silica gel grids were used to locate the position of electrodes on the face. The grid on the forehead had 10 columns and three rows. The grid on the right and left sides of the cheek had five columns and six rows. The inter-electrode distance (IED) was 15 mm. **(B)** Illustration of a subject with high-density electrodes. **(C)** Illustration of face muscles covered by electrodes.

The surface myoelectric activity of facial muscles was obtained using a High-density surface Electromyography (HD sEMG) recording system (TMSi, REFA 128-model, the Netherlands, sampling frequency 2,048 Hz). Up to 90 channels of monopolar EMG signals were simultaneously recorded during the facial motor tasks. Also, the 90 electrodes were divided into three subsets, which covered the forehead, right cheek, and left cheek regions, respectively. Each subset had 30 electrodes arranged in a rectangular array. The subset that covered the forehead was arranged in three rows and 10 columns while the ones that covered both sides of the cheek were arranged in six rows and five columns. Before placement of the electrodes, facial skin was cleaned using alcohol cotton stickers, then marked according to a pre-punched template. The template was used for arranging electrodes as mentioned above. The locations of templates were determined according to the following two simple criteria: (1) the location of the lower edge of the forehead template was at the upper edge of the eyebrow; and (2) the locations of the medial edge of the cheek templates were at the vertical line of mouth corner. The distance between the punch holes on the template was 15 mm. Illustrations of the template and placement of the electrodes on the face were shown in Figure 1. The reference electrode was placed on the carpal bone area.

### Experimental Procedure

**Figure 2:**
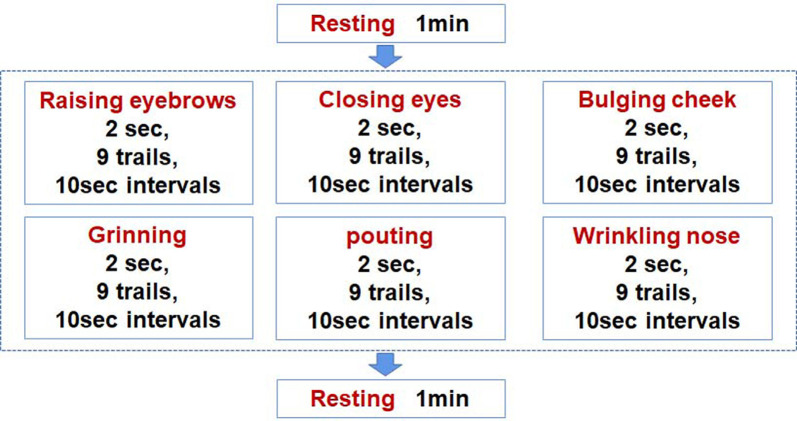
Illustration of the experimental procedure.

Six different facial motor tasks, including RE, CE, BC, GR, PO, and WN, were first demonstrated to the subjects. Afterward, the subjects practiced the demonstrated motor tasks until they were considered fit to complete the tasks in a standardized manner. The subjects were instructed to complete the movement with maximum effort to make sure the activation level of facial muscles was almost the same among different subjects. Thereafter, the electrodes were attached to the subjects’ faces after which they were verbally guided to complete the six facial movements. Each movement lasted for 2 s and was repeated nine times at an interval of 10 s. Before the tasks, subjects were asked to sit upright and keep still for 1 min. During the experiment, the EMG amplitude was monitored in real-time to ensure that the subjects completed the tasks as required. An illustration of the experimental procedure is shown in Figure 2.

### Signal Processing and Data Analysis

The monopolar signals were offline bandpass filtered using a zero-phase fourth-order Butterworth filter (20–500 Hz) to remove lower frequency movement artifacts and high-frequency noises. Additionally, a 50 Hz notch filter was used to remove power line noise.

For each motor task, the filtered EMG signals comprised of nine active segments corresponding to nine repeated trials of muscle contraction. To avoid the inclusion of EMG signals of the non-activated segment, the onset and offset times of each active segment were manually determined according to the sudden increase and decrease of the amplitude of the EMG signal and applied to all channels. For each channel, the RMS value of every active segment was calculated to represent muscle activity during the motor task. RMS was calculated using equation 1.

(1) $$\text{RMS}=\sqrt{\frac{\sum_{i=1}^{N}x_{i}^{\text{2}}}{N}}$$

**Figure 3:**
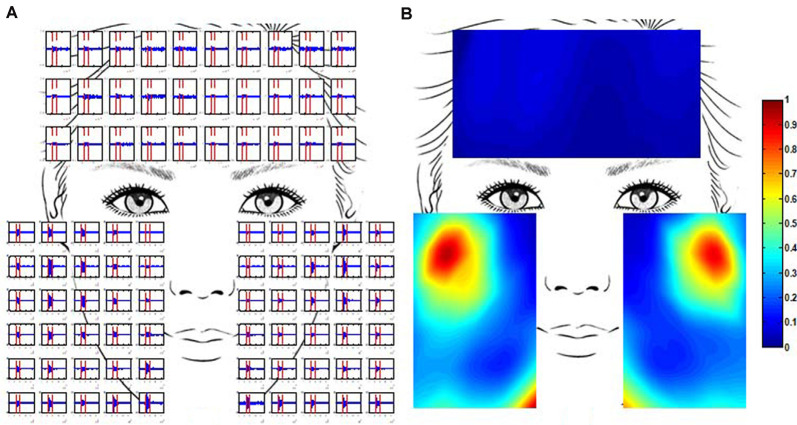
Multi-channel electromyogram (EMG) recording and the construction of EMG maps. **(A)** Multi-channel EMG signals from a healthy subject were simultaneously recorded using the high-density surface EMG (HD sEMG) system. EMG signals between two vertical red lines were obtained in the course of grinning (GR). **(B)** The activation map of the same healthy subject in the course of GR. The map was constructed from the root mean square (RMS) of multi-channel EMG signals. The RMS values were normalized to the maximum value.

Where *N* was the total number of sample data in the selected segment and *x_i_* was the amplitude of the *i*^th^ sample data. The multi-channel RMS values for each trail were then normalized to the difference between the maximum and minimum RMS values for the trail. This is because the amplitude range of the recorded EMG signals varied at each trail. The activity of all the facial muscles during an active segment could be shown simultaneously on a topographic map which was constructed from the normalized multi-channel RMS values using a cubic interpolation algorithm. Figure 3 illustrates how the EMG topographic map was obtained from the active segments of the multi-channel EMG signals. The hot (toward red) color represents a high RMS value while the cold (toward blue) color indicates a low RMS value.

The EMG maps represent the spatial distribution of activated muscles. The maps enable researchers to observe the position and shape of activated facial muscles during motor tasks. A comparison of muscle activation patterns among different tasks was then made accordingly. Additionally, a comparison between healthy and Bell’s palsy subjects during different motor tasks was made. Thereafter, the similarity in muscle activation patterns between the two sides of a face was quantitatively evaluated using the 2D correlation coefficient (corr2), which was calculated as equation (2).

(2) $$\text{corr2}=\frac{\sum_{m}\sum_{n}\left(L_{mn}-\bar{L}\right)\left(R_{mn}-\bar{R}\right)}{\left(\sum_{m}\sum_{n}{\left(L_{mn}-\bar{L}\right)}^{\text{2}}\right)\left(\sum_{m}\sum_{n}{\left(R_{mn}-\bar{R}\right)}^{\text{2}}\right)}$$

Where corr2 was the 2D correlation coefficient while *L* and *R* depicted the EMG mapping data matrices of the left and right sides of a face, respectively. m and n represented the number of lines and columns in the matrices, respectively. The larger the value of corr2, the stronger the correlation was between the two sides of facial activities. Values of 1 and −1 depicted a completely positive and negative correlation, respectively, whereas 0 indicated no correlation. The corr2 of the forehead and cheek were calculated separately.

To quantitatively analyze the location of activated muscles, a spatial feature—Center of Gravity (CG) was extracted based on the EMG maps. First, the EMG maps were segmented based on the threshold method where an experience threshold of 0.5 was set to get the region of activated muscles. Afterward, the CG coordinates were calculated as equations (3) and (4).

(3) $${\text{CG}}_{x}=\frac{\text{1}}{\sum_{i,j}Map_{i,j}}\sum_{i,j}Map_{i,j}*j$$

(4) $${\text{CG}}_{y}=\frac{\text{1}}{\sum_{i,j}Map_{i,j}}\sum_{i,j}Map_{i,j}*i$$

Where CG_*x*_ and CG_*y*_ representing the CG coordinates in the *X* and *Y* directions. *Map_i,j_* represented the *i* × *j*th element in the segmented EMG map while *i* and *j* represented the line and column position of the *i* × *j*th element.

The CGs of both sides of the forehead and cheek were calculated separately. Moreover, the CG distance was defined and calculated to quantitively compare the location of facial muscle activation between two sides of a face. The CG distance was defined as the length between the right CG and left CG after mirroring the right CG to the left part of the forehead or cheek map.

### Statistics

Comparison of corr2 in forehead and cheek region between healthy and Bell’s palsy subjects during different tasks were tested using the independent sample *t*-test. Additionally, Pearson’s correlation coefficient between the corr2 and FNGS scores was calculated. The correlation coefficients in the forehead and cheek were calculated separately. The FNGS scores included a series of regional scores such as the brow, eye, nasolabial fold (NLF), and oral scores. The sum of these regional scores was considered as the total score. The correlation between corr2 and each score was evaluated. Besides, a comparison of CG distance in forehead and cheek region between healthy and Bell’s palsy subjects during different tasks were also tested using the Wilcoxon rank-sum test since the sample sizes of these two groups were not always the same. A *p*-value of less than 0.05 was considered statistically significant.

## Results

### The Activation Patterns of Facial Muscles in Healthy and Bell’s Palsy Subjects

**Figure 4:**
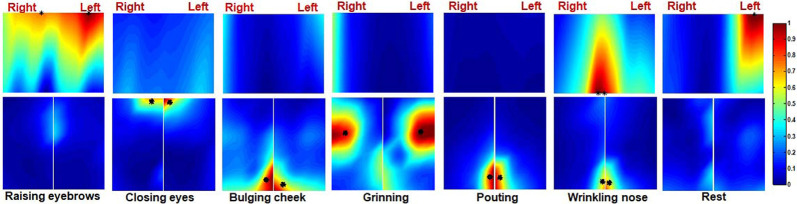
The activation map of a healthy subject during six different facial movements. The centre of gravity (CG) of the left and right side of the map is also marked (*).

Ten healthy subjects were recruited in this study and their ID numbers ranged from 11 to 20. The EMG maps showed that patterns of muscle activation during tasks were nearly symmetrical in healthy subjects. For instance, the EMG maps of subject 15 during six different facial motor tasks were shown in Figure 4. The frontalis was mainly recruited by RE therefore the top region of the forehead subset map was highlighted. On the other hand, CE mainly recruited the orbicularis oculi so regions near the eyes were highlighted on both the forehead and cheek subset maps. Moreover, BC mainly recruited the orbicularis orris therefore regions near the mouth were highlighted on the left and right cheek subset maps. The zygomaticus was mainly recruited by GR so the central regions of the left and right cheek subset maps were highlighted. The EMG maps of PO were somewhat similar to those of BC. Furthermore, WN mainly recruited the corrugator and nasalis, so the median region of both the forehead and cheek subset maps were highlighted. Finally, CG was symmetrically distributed on the EMG maps as shown in Figure 4.

**Figure 5:**
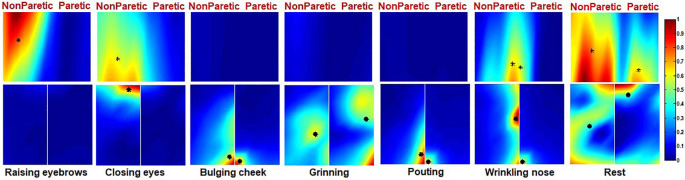
The activation map of a Bell’s palsy subject during six different facial movements. The CG of the left and right side of the map is also marked (*).

Ten Bell’s palsy subjects were recruited in the study with ID numbers ranging from 1 to 10. The extent of their condition was assessed using the FNGS 2.0, as shown in Table 1. The EMG maps revealed that patterns of muscle activation were asymmetric in Bell’s palsy subjects. Figure 5 showed the EMG maps of subject five during six different tasks. The subject was a Bell’s palsy paralyzed on the left. In this subject, only the frontalis on the right side was highlighted during RE. However, when the subject was performing CE, both the frontal and orbicularis oculi on the right side of the face were highlighted. Additionally, the orbicularis orris on both sides of the face was highlighted during BC although the marked area on the healthy (right) side was larger than that on the paretic (left) side. During GR, the zygomaticus on both sides of the face was highlighted although they were distributed asymmetrically. Notably, the masseter on the paretic side of the face also seemed to be recruited during GR since the bottom left corner of the left cheek map was highlighted. Moreover, the orbicularis orris on both sides of the face was recruited during PO, similar to BC. Finally, WN recruited both the corrugator and nasalis. The results revealed that the highlighted area on the healthy side was larger than the paretic side during PO and WN. The CGs were also labeled in Figure 5.

The EMG maps during rest are also shown in Figures 4, 5. Although the amplitude of the EMG signals was weak during rest, there was an uneven distribution of electrical activities in facial muscles. It seemed that the electrical activities in healthy representative subjects were more symmetrical than those in Bell’s palsy representative subjects during rest. However, the spatial distribution patterns of facial EMG during rest were diverse in different subjects. Therefore, spatial characteristics of EMG maps during rest, such as corr2 and CG, will not be extracted and analyzed in the next sections.

### The Similarity of Muscle Activation Patterns Between Two Sides of the Face

**Figure 6:**
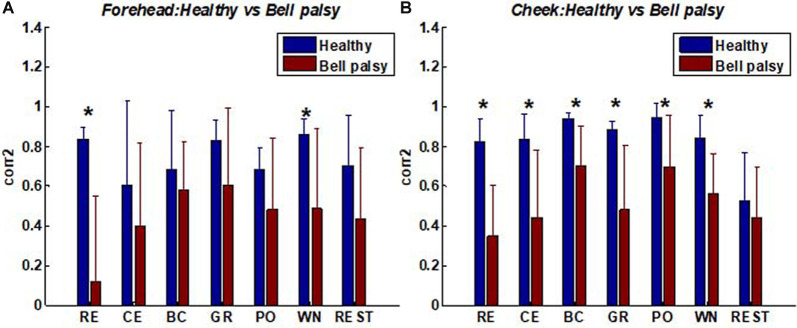
Average corr2 of healthy and Bell’s palsy subjects in different tasks. **(A)** The average corr2 in the forehead region; **(B)** the average corr2 in the cheek region. Raising Eyebrow (RE), Closing Eyes (CE), Bulging Cheek (BC), Grinning (GR), Pouting (PO), and Wrinkling Nose (WN). Error bars represent the standard deviation between subjects (*p < 0.05).

Moreover, the similarity in the patterns of facial muscle activation was evaluated using corr2. A comparison of corr2 between healthy and Bell’s palsy subjects is shown in Figure 6. The figure shows that the mean corr2 values of healthy subjects were higher than those of Bell’s palsy victims during all the tasks as well as during rest. Significant differences were particularly observed in the forehead region during RE and WN and in the cheek region during all the tasks.

The correlation coefficients between corr2 and FNGS scores are shown in Tables s 2, 3. In these tables, *p* values of correlation that were smaller than 0.05 and 0.01 were marked, respectively. Table 2 shows that the correlation between corr2 and FNGS scores (except Eye score) during RE and CE was more significant in the forehead region since the *p* values were smaller than 0.01. Also, there was a significant correlation between corr2 and FNGS scores during GR and WN since *p* < 0.05 were obtained. On the other hand, Table 3 shows that there was a significant correlation between corr2 and FNGS scores (except Eye score) during BC and PO in the cheek region (*p* < 0.05). No significant correlation was observed between corr2 and FNGS scores in both the forehead and cheek regions during GR, WN, and rest. These results showed that the symmetry of facial EMG activities in Bell’s palsy patients was significantly lower than that in healthy subjects, and the spatial symmetric feature corr2 of some facial EMG map regions has the potential to predict the condition of Bell’s palsy during some tasks.

**Table 2:** The correlation coefficients between forehead 2D correlation coefficient (corr2) and Facial Nerve Grading Scale (FNGS) scores during different tasks.

FNGS score		RE	CE	BC	GR	PO	WN	REST
Brow	R	−0.9533	−0.8214	−0.0457	−0.5225	−0.2122	−0.3118	−0.2944
	P	0.0000**	0.0036**	0.9002	0.1213	0.5561	0.3804	0.4090
Eye	R	−0.6087	−0.6827	−0.1370	−0.7908	−0.4868	−0.2285	−0.3110
	P	0.0618	0.0296*	0.7060	0.0064**	0.1536	0.5255	0.3818
NLF	R	−0.8133	−0.8514	−0.4286	−0.6334	−0.4068	−0.4345	−0.1393
	P	0.0042**	0.0018**	0.2166	0.0493*	0.2433	0.2096	0.7011
Oral	R	−0.8864	−0.8873	−0.3120	−0.5613	−0.2780	−0.4172	−0.0621
	P	0.0000**	0.0000**	0.3801	0.0914	0.4367	0.2303	0.8646
Total	R	−0.9208	−0.8746	−0.2066	−0.6230	−0.3267	−0.3765	−0.2299
	P	0.0000**	0.0000**	0.5669	0.0543	0.3568	0.2835	0.5229

R is the correlation coefficient; P indicates statistical significance (*P < 0.05; **P < 0.01).

**Table 3:** The correlation coefficients between cheek corr2 and FNGS scores during different tasks.

FNGS score		RE	CE	BC	GR	PO	WN	REST
Brow	R	0.3244	−0.2486	−0.6916	−0.4334	−0.7572	−0.2870	0.2007
	P	0.3605	0.4885	0.0267*	0.2109	0.0112*	0.4213	0.5782
Eye	R	0.1841	−0.4618	−0.7324	−0.3969	−0.5439	−0.3626	0.1061
	P	0.6106	0.1791	0.0160*	0.2561	0.1041	0.3032	0.7705
NLF	R	0.3656	−0.3376	−0.7751	−0.5578	−0.6524	−0.2958	−0.0426
	P	0.2988	0.3400	0.0085**	0.0938	0.0409*	0.4066	0.9071
Oral	R	0.3239	−0.2704	−0.7977	−0.5830	−0.7110	−0.3512	−0.0898
	P	0.3613	0.4498	0.0057**	0.0769	0.0211*	0.3198	0.8052
Total	R	0.3226	−0.3190	−0.7857	−0.5050	−0.7426	−0.3210	0.1383
	P	0.3632	0.3690	0.0071**	0.1365	0.0139*	0.3659	0.7032

R is the correlation coefficient; P indicates statistical significance (*P < 0.05; **P < 0.01).

### The Distribution of CGs During Different Tasks

**Figure 7:**
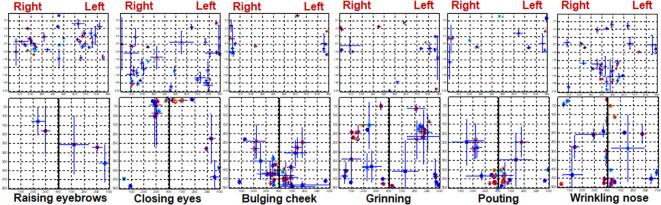
The distribution of CGs during different tasks. The location of each subject’s CG is marked on the rectangular coordinate system. The CGs of healthy subjects are indicated in red while those of Bell’s palsy victims are indicated in blue. The vertical error bars represent the standard deviation of *Y* coordinates between trails. Horizontal error bars represent the standard deviation of *X* coordinates between trails.

CGs can best represent the center position of activated muscles if the highlighted area is segmented accurately. In this study, multiple thresholds were tested and 0.5 was shown to be able to effectively segment most maps. The CGs were extracted from the segmented maps and CGs of each trail were inspected carefully. The mean distribution of CGs in every task is shown in Figure 7. The CGs of healthy and Bell’s palsy subjects were labeled in red and blue respectively. Also, different subjects were labeled with different shades of red or blue, respectively. The standard deviation of *X* and *Y* coordinates of each CG was plotted at the position of CG horizontally and vertically.

Figure 7 shows that CGs of the forehead were mainly distributed in the middle area in healthy subjects while those of Bell’s palsy victims were distributed away from the middle line. Additionally, CGs of the right and left cheeks were consistent with the position of the activated muscles during facial movement. During RE, the cheek muscles were almost stationary so fewer CGs were extracted from the cheek region. Moreover, the orbicularis orris contracted during BC and PO therefore the CGs were mostly distributed at the bottom midline corner. During CE, the orbicularis oculi contracted so CGs were mostly distributed at the upper midline corner. Furthermore, CGs were mostly distributed in the central area of the cheek during GR while in WN they were majorly distributed near the midline between right and left cheeks. It is noteworthy that the CGs of healthy subjects were more symmetrically distributed compared to those of Bell’s palsy victims regardless of the paretic side.

### The CG Distance Between Two Sides of the Face

The location of the CG is related to the position of facial muscle activation. It can be observed from Figure 7 that CGs of the paralyzed side of the face were not symmetrically distributed with the position of the CGs on the healthy side. Therefore, a spatial feature of the EMG map called CG distance was extracted to quantify asymmetry in the location of activated muscles between two sides of the face. The CG distance of the forehead and cheek were calculated separately and were described in detail in section Signal processing and data analysis.

**Figure 8:**
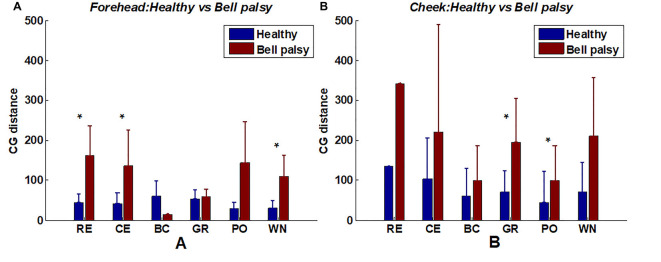
Average CG distance of healthy and Bell’s palsy subjects in different tasks. **(A)** The average CG distance in the forehead region; **(B)** the average CG distance in the cheek region. Error bars represent the standard deviation between subjects (*p < 0.05).

A comparison of CG distance between healthy and Bell’s palsy subjects is shown in Figure 8. The figure shows that the mean CG distance of healthy subjects was lower than those of Bell’s palsy victims during most tasks. Unlike corr2, CGs cannot be extracted if the intensity of some EMG map regions were lower than the threshold value of 0.5. Thus, some subjects’ CG distance data was missing in the process of extracting CG distance. Therefore, the Wilcoxon rank-sum test was used to test the significant difference of mean CG distance between healthy subjects and Bell’s palsy victims since the sample sizes of these two groups were not always the same. Significant differences were particularly observed in the forehead region during RE, CE, and WN and in the cheek region during GR and PO.

It is noteworthy that RE, CE, and WN recruited the forehead muscles while BC, GR, and PO recruited the cheek muscles. It indicated that in the position of activated facial muscles, the CG distances of Bell’s palsy subjects tended to be larger significantly than that of healthy subjects.

## Discussion

In this study, the HD sEMG method was used to simultaneously record the distribution of facial muscle activities during six facial movements and rest. Consequently, electrode grids were used to cover the forehead and cheek regions of the face, therefore, no prior knowledge of facial anatomy was needed for the placement of the electrodes. This was different from the multi-channel surface EMG study on facial muscle activation patterns (Schumann et al., 2010). Additionally, Bell’s palsy subjects were recruited in the present study of the measurement of facial muscle activation through EMG. The study aimed to acquire the activation patterns of facial muscles in both healthy and Bell’s palsy subjects. The regions of activation were obtained during six frequently used facial movements for the assessment of Bell’s palsy. The movements included RE, CE, BC, GR, PO, and WN. The results revealed that the activation patterns in the facial muscles of healthy subjects were more symmetric compared to those in Bell’s palsy victims. This was quantified using the corr2 and distance on the left and right sides of the CG. Corr2 was also correlated with Bell’s palsy, which was assessed using the FNGS scale. Overall, the computed spatial muscle activation maps provided detailed information on the activity of facial muscles during facial movement. This was related to the physiological and pathological background of the face.

### The Number and Localization of High-Density Electrodes

Facial muscles, characterized by high heterogeneity, are a group of skeletal muscles innervated by the facial nerve (Gilden, 2004). Knowledge of facial muscles is vital in understanding the physiological process of diverse facial movements. Except for a few studies focusing on the anatomy of facial muscles (Odwyer et al., 1981; D’Andrea and Barbaix, 2006; D’Antoni, 2016), the electrophysiological method are frequently used in the study of these muscles (Grosheva et al., 2008; Lapatki et al., 2010; Han et al., 2015; Kim et al., 2018). However, too few electrodes are used in a majority of these electrophysiological studies therefore the activation of multiple facial muscles cannot be detected simultaneously. Consequently, a non-invasive and multi-channel surface EMG method can be used to obtain the activation patterns of several facial muscles. A previous study by Lapatki’s et al. recorded (Lapatki et al., 2003, 2006, 2010) the topographical activation of perioral muscles such as depressor angular orris and orbicularis orris inferior using HD sEMG grids (120 channels) with an interelectrode distance of 4 mm. Therefore, the location, spatial orientation, and innervation zone of facial musculature could be analyzed through this High-density EMG signal recording method (Lapatki et al., 2006). Nonetheless, the electrode grids used in this study were too dense such that if the activation of muscles in the entire face was to be recorded simultaneously, thousands of electrodes would have to be utilized yet no HD sEMG hardware system can support this application. Moreover, Schumann et al. (2010) attached 48 channels of surface electrodes to the face to simultaneously record the activities of the main facial muscles (Schumann et al., 2010). The position of the electrodes was determined by the anatomical knowledge of facial muscles which may be varied across different subjects (Cattaneo and Pavesi, 2014).

In this study, a less dense electrode grid with 90 electrodes was used to nearly cover the entire face. The localization of electrodes did not need prior knowledge of the anatomical position of facial muscles, therefore reducing the deviation of EMG signals caused by the subjective selection of electrode position. The diameter of the electrodes was about 5 mm and the Inter-electrode Distance (IED) was 15 mm. Both bipolar differential (Ferraioli and Armando, 1986; Roeleveld and Stegeman, 2002) and monopolar EMG signals can be obtained from the HD sEMG signals. In this study, monopolar EMG signals were used to represent muscle activations for two reasons: (i) the monopolar EMG signal reflects both superficial and deep EMG sources while the bipolar EMG signal only reveals superficial EMG sources (Scholle et al., 2001). Considering the largely overlapping facial muscles 4, the monopolar EMG signal could preserve more features of facial muscles despite the increase in crosstalk; and (ii) given the various myofiber orientations of facial muscles (Cattaneo and Pavesi, 2014), the spatially filtered EMG signals such as bipolar differential EMG signals could have exerted a differential effect in different locations of facial muscles (Roberto and Parker, 2004). Also, studies with a similar configuration obtained spatially distributed EMG maps of neck muscles during swallowing (Zhu et al., 2017). The monopolar EMG maps showed in this study contributed to the basic knowledge of the activity of facial muscles during different facial movements.

### Distinct Facial Muscle Activation Patterns During Different Tasks

It was reported that the mean EMG amplitudes of the orbicularis oculi and frontalis muscles were lower than those of the perioral muscles (Schumann et al., 2010). The EMG amplitudes of different subjects also varied. In this study, the EMG signals were normalized to the maximum EMG amplitude, therefore, the HD sEMG map reflected the distribution of relative intensity in EMG signals.

During RE, the forehead muscles were activated and the CGs of the forehead EMG map was located at the top half part as shown in Figures 4, 5, 7. In the monopolar EMG map, the location of signals with the highest amplitude corresponded to the main innervation zone of the muscle. Also, the highest amplitude signal was progressively lower along the muscle fiber (Kleine, 2000). This means that the activated frontalis muscle was mainly distributed on the top of the forehead during RE. A previous study did not report the activated region of the frontalis muscle since the electrodes were only attached to the lower part of the muscle (Schumann et al., 2010). Moreover, muscles near the eyes, including the corrugator and orbicularis oculi, were activated during CE. However, closing the eyelids does not require a lot of force, and only forced closure of the eyelids can produce a notable activation of periorbital muscles. The results also showed that RE and CE did not largely recruit muscles in the cheek region.

BC, GR, and PO mainly recruited muscles in the cheek region, but not those in the forehead. Also, the facial activation patterns during BC and PO were similar and both activated the orbicularis orris muscle, which was consistent with the results obtained by Schumann et al. (2010) and Cacaou et al. (1996). It was reported that numerous small innervation zones are scattered over the entire surface of the orbicularis orris muscle (Lander et al., 1996), therefore, a variety of shapes in the mouth can be produced during the contraction of this muscle. In this study, both BC and PO needed the subjects to purse their lips as well as blow out their cheeks. Both movements displayed almost similar activation patterns. Completing the GR movement also needed the variation of the orbicularis orris muscle, although the main activated muscle in this study was the zygomaticus, consistent with the results obtained by (Schumann et al., 2010). Moreover, GR also involved pulling the corners of the mouth up and back, which can be seen as a slight activation of muscles near the corners of the mouth. On the other hand, WN recruited muscles in both the forehead and cheek regions. Standard activation patterns of this movement include the activation of pyramidalis nasi and nasalis. It was reported that the elevator angular orris muscles might also be active during WN, although activation of the muscles had little contribution to the sEMG signals since they are located in a deeper tissue layer (Lapatki et al., 2003). In this study, there was an obvious activation of the elevator angular orris as can be seen in the monopolar EMG map shown in Figure 4.

Compared to previous studies utilizing surface EMG techniques in recording facial muscle activities (Schumann et al., 2010; Kim et al., 2018), this study is the first one to report the activation patterns of facial muscles in Bell’s palsy subjects using the HD sEMG method. The activation patterns in Bell’s palsy displayed two characteristics: (i) the amplitude of the EMG signals on the affected side of the face was decreased; and (ii) there was an asymmetric distribution of the activated regions of facial muscles during facial movements. Figure 5 shows the asymmetric EMG maps of Bell’s palsy subjects. The asymmetry might have occurred due to three reasons. One was the absence of EMG activities during RE, CE, and WN as shown in the figure. A second reason might have been due to the reduced activated area during BC and PO. The third reason was due to the shift in the location of the activated region during GR. Significant differences in EMG amplitude between paralyzed and non-paralyzed sides of the face have been reported previously (Wenceslau et al., 2016). This characteristic highlighted why facial muscles on the paralyzed side were weaker in Bell’s palsy. However, it is not clear why there was a shift in the activated region on the paralyzed side of the face. The electrodes used in this study were not connected by a grid, contrary to the thin and flexible electrode grid used by Lapatki et al. (2006, 2010). They were attached separately on the face. Therefore, the shift in the activated region should not be affected by the drift between electrodes and skin surface during muscle contraction. One possible explanation for this phenomenon is the shortening of muscles during contraction (Huang et al., 2019). The weakness of paralyzed facial muscles led to a different degree of muscle contraction force compared to the non-paralyzed side. Consequently, the activated regions were distributed differently between the two sides of the face.

### The Correlation Between Facial Muscle Activation Features and Bell’s Palsy

Two features were extracted from the facial muscle activation maps, namely the 2D correlation coefficient (corr2) and Center of Gravity (CG). Corr2 quantified the symmetry of EMG activities between two sides of the face. On the other hand, the CG was extracted from the segmented EMG map and represented the centroid location of the activated muscle region. Additionally, Bell’s palsy was assessed through a commonly used scale that incorporated regional scoring of facial movement, called the FNGS 2.0. Assessing Bell’s palsy subjects using FNGS 2.0 require patients to make a series of facial movements after which the examiner rates the movement in each of the following four areas: brow, eye, nasolabial fold (NLF), and oral commissure. In this study, subjects were asked to make facial movements, including RE, CE, BC, GR, PO, and WN. These movements are frequently used in the clinical assessment of Bell’s palsy. Afterward, the comparison of corr2 and CG distance between healthy and Bell’s palsy subjects was conducted, and the correlation between corr2 and Bell’s palsy was evaluated to reveal the connection between facial neuromuscular activities and the subjective observation of the examiner.

It had been found in this study that both corr2 and CG distance of the activated facial muscle region (forehead or cheek) were different significantly between healthy and Bell’s palsy subjects. The corr2 and CG distance were high in the forehead region for tasks such as RE, CE, and WN that mainly recruited the forehead muscles. Also, the corr2 and CG distance were high in the cheek region during tasks such as BC, GR, and PO, which mainly recruited cheek muscles. To further explore whether these spatial features were related to Bell’s palsy, a correlation analysis between corr2 and FNGS scores was conducted. The results showed that the significance of the correlation between corr2 and FNGS scores was related to the type of facial movement. The correlation between corr2 and Bell’s palsy was significant under the facial muscle regions where the task activated. Although the correlation between facial expression and facial EMG has previously been investigated (Wolf et al., 2005; Jiang et al., 2015), the association of facial movements and their EMG signals in Bell’s palsy was first reported in this study. These results demonstrated the connection between the activity of facial muscles and changes in the superficial geometry of the face (Cattaneo and Pavesi, 2014). The connection between facial activities and facial geometry also helped in explaining why the function of the facial nerve can be assessed automatically from visual face capture using image processing, computer vision, and machine learning techniques (Guarin et al., 2018; Johnston and De Chazal, 2018; Lou et al., 2020).

It is noteworthy that there was no significant difference in the correlation coefficients of different FNGS scores for the same task. Initially, the study hypothesized that the correlation coefficients of the eye scores during a specific task like CE would be more significant than those of the other scores. However, the results disproved this hypothesis. One possible explanation of this observation may be because the impaired segment of the facial nerve in Bell’s palsy was located inside the skull (Peitersen, 2002) therefore muscles on the paralyzed side of the face were affected to the same extent. Also, the significant differences among the various regional scores may have existed in the sequel of Bell’s palsy (Gilden, 2004). However, Bell’s palsy subjects recruited in this study had an onset time of between 0 and 3 months, at which the sequels could not happen (Wenceslau et al., 2016).

### Limitations of the Study

Despite the insightful findings, this study had several limitations. First, the electrodes attached to the face were placed according to the grid labeled in advance on the skin surface. Also, the electrodes were placed in a rectangular fashion and therefore did not cover some corner regions of the face such as the eyelids, corner of the mouth, and nose wing. As a result, the muscle activation patterns in these regions could not be shown during facial movements. Second, the size of each subjects’ face was different. As a result, the location of activated muscle regions in each participant was inconsistent. However, relative values such as the corr2 and the CG distance of two sides of the face were used to minimize the differences between individuals. Third, the distance between the electrodes was relatively large (15 mm) compared to other studies investigating regional facial muscle activities (Lapatki et al., 2006, 2010). Moreover, the analysis of EMG signals at the motor unit level could not be conducted due to the spatial resolution of EMG grids. Nonetheless, the activation patterns of the whole face and its spatial features such as corr2 and CG could be acquired based on the configuration of these electrodes. Finally, this pilot study contained a relatively small number of Bell’s palsy subjects. Therefore, some correlation analysis such as the correlation between CG distance and FNGS scores was meaningless and was not shown in the result. Therefore, more Bell’s palsy participants with different manifestations and onset times need to be included in future studies.

### Implications

Despite the crosstalk of monopolar EMG signals, the EMG maps were still precise enough to show the distinct activation patterns in facial muscles during different facial movements. Also, the amplitude of activated facial EMG signals was high despite the highest EMG activity did not correspond to the size of the entire muscle (Kleine, 2000). From a physiological point of view, the action potential of a muscle is generated at the innervation zone, propagates along the sarcolemma, and disappears at the tendons. Therefore, regions of a summit on the EMG map at high intensity will show the main innervation zone of the activated muscles (Roberto and Parker, 2004). The precise examination of the main innervation zone of facial muscles will not only help physicians in assessing the condition and location of impaired facial muscles but also guide the physical treatment of Bell’s palsy (Ordahan and Karahan, 2017).

Also, the HD sEMG used in this study depicted the selective activation map of the facial muscles. Given the complex structure and function of the facial motor system (Cattaneo and Pavesi, 2014), the HD sEMG method may potentially be useful in research on the physiological coactivation of facial muscles (Schumann et al., 2010). This method is also advanced since no prior anatomical knowledge of facial muscles is needed for the attachment of electrodes. Moving forward, the EMG maps will show where the muscles are activated, allowing researchers to find the anatomical position of the activated muscles through the labels of electrodes on the face.

## Conclusions

This study quantified the spatial activation patterns of both the forehead and cheek region of the face during different facial movements. The facial activation EMG maps constructed from the HD sMEG signals provides a means of localizing the activated muscles and this can be helpful in the diagnosis as well as treatment of facial neuromuscular diseases such as Bell’s palsy. Furthermore, the correlation between features of spatial activation patterns and Bell’s palsy was analyzed in this study. The significant correlation between the objective EMG features and subjective scores indicated the close connection between facial muscle activities and the geometric changes on the surface of the face. In summary, the facial muscle activation patterns in both healthy and Bell’s palsy subjects are useful in understanding facial muscle disorders. We, therefore, suppose that the HD sEMG technique can provide more information on facial muscle disorders at the motor unit level in the future.

## Data Availability Statement

The raw data supporting the conclusions of this article will be made available by the authors, without undue reservation.

## Ethics Statement

The studies involving human participants were reviewed and approved by Institutional Review Board of Shenzhen Traditional Chinese Medicine Hospital (Approval Number: 2017-05). The patients/participants provided their written informed consent to participate in this study. Written informed consent was obtained from the individual(s) for the publication of any potentially identifiable images or data included in this article.

## Author Contributions

HC, WZ and HY conceived the presented idea. HC and GL developed the methods of high-density EMG signal analysis and performed the computations. ZY and XC were in charge of recruiting patients. SD carried out the experiments. LH and KL were in charge of recruiting healthy participants. XH supervised the findings of this work. HC took the lead in writing the manuscript. All authors contributed to the article and approved the submitted version.

## Conflict of Interest

The authors declare that the research was conducted in the absence of any commercial or financial relationships that could be construed as a potential conflict of interest.
